# Comparative transcriptome analysis reveals key candidate genes mediating ovarian development in *Spodoptera frugiperda* fed on two host plants

**DOI:** 10.3389/fphys.2022.1056540

**Published:** 2022-11-15

**Authors:** Renwen Zheng, Ling Yao, Jun Peng, Zihan Chen, Fan Yang, Shuxian Chen, Qingfeng Tang

**Affiliations:** Anhui Province Key Laboratory of Integrated Pest Management on Crops, Key Laboratory of Biology and Sustainable Management of Plant Diseases and Pests of Anhui Higher Education Institutes, School of Plant Protection, Anhui Agricultural University, Hefei, China

**Keywords:** *Spodoptera frugiperda*, ovary, host plants, ovary development-related genes, plant-insect associations

## Abstract

The fall armyworm (FAW), *Spodoptera frugiperda*, is a highly polyphagous lepidopteran pest, with its growth and adaptation affected by different host plants. However, little is known about the effects of host plants on ovarian development in this species. Thus, we evaluated the effects of feeding on corn (*Zea mays L.*) and goosegrass (*Eleusine indica*), on the ovarian development of *S. frugiperda*. Using various stages of *S. frugiperda*, we also evaluated the larval and pupal weights, number of eggs, and differentiation of ovarioles over time. Results showed that females fed on goosegrass had shorter ovarioles and laid less eggs than those fed on corn. Transcriptome analysis identified 3,213 genes involved in ovarian development in the fall armyworm. Of these, 881 genes were differentially expressed when fed on corn and goosegrass. The analysis also indicated that the hormone biosynthetic pathways may be involved in the reproductive system. In relation to the reproductive function, nine juvenile hormone (JH) biosynthetic genes, four 20-hydroxyecdysone (20E) biosynthetic genes, and four ovary-relevant functional genes were identified. The time course of the expression profiles of these hormone- and ovary development-related genes was measured by quantitative real-time PCR (qRT-PCR). In total, six of them showed a decreasing trend in the ovary of the FAW fed on goosegrass, while two genes showed an increasing trend. Our results showed that significant changes in the reproductive activity/ovary development in the FAW occurred in response to different diets. These results serve as bases for evaluating how optimal host plants and feeding preference affect ovarian development in the FAW.

## Introduction

The lepidopteran pest, the fall armyworm (FAW), *Spodoptera frugiperda* (J. E. Smith), is an important polyphagous insect pest that causes serious economic threats to crops and increases its population all year round. Native to the Western Hemisphere, the FAW was first detected on the African continent in 2016 and has subsequently spread throughout the continent and across Asia (Harrison et al., 2019). *S. frugiperda* is one of the most important insect pests of corn and prefers to feed on leaves and tender shoots during the larval stage. The FAW is also a herbivore of 353 plants species, found in 227 genera, and 76 families (Paredes-Sanchez et al., 2021). Its damage to maize, rice, sorghum, and sugarcane has been estimated to be about US$13 billion per annum in crop losses across Africa (Rwomushana et al., 2018). The FAW is difficult to control and manage since it is polyphagous and trans-boundary, multiplies fast, has a short life cycle and travels over long distances, and does not enter diapause (Matova et al., 2020). Therefore, there is an urgent need to develop effective green management strategies to minimize its damage to crops.

Insects are exposed to changes in external factors throughout their life cycle. When confronted with these changes, they adjust their development and physiology to ensure that they can produce the functional structures necessary for survival and reproduction (Mirth et al., 2021). Development and reproduction are strongly affected by the quality of host plants (Fang et al., 2021). Different host plant species have large variations in their nutritive values and metabolite composition that may affect insects’ fitness, development, and reproduction (Schillewaert et al., 2017). The availability of different host plants is closely related to the growth, development, and outbreaks of polyphagous insect populations. The quality and quantity of host plants fed on by insects may affect their growth, development, physiology, and reproduction (Scriber and Slansky, 1981). Meanwhile, the use of host plant metabolites to control insect pests is a major part of integrated pest management (IPM) (Hemati et al., 2012). This approach is simple, convenient, and green, and is compatible with other methods of pest control. Development of resistant cultivars against FAW attacks would serve as an effective complementary approach in IPM to reduce its damage levels (Jallow et al., 2004). Aryl sugars on the surface of leaves are a powerful barrier against some insects, as shown by the ability of *Bemisia tabaci* (whiteflies) to reproduce on *Nicotiana benthamiana* with an acylsugar acyltransferase knockout but not on wildtype plants (Feng et al., 2021). However, the contributions of host plants to the reproductive system in *S. frugiperda* are not clear.

The FAW undergoes complete metamorphosis, through larval–pupal–adult stages. It becomes sexually mature and is capable of reproductive activity at the adult stage. Insect metamorphosis and reproduction are governed by two critical hormones, juvenile hormone (JH) and 20-hydroxyecdysone (20E) (Song and Zhou, 2020), which are well known to play a gonadotropic role in adult insects (Gruntenko and Rauschenbach, 2008). In larval development, 20E mediates larval–pupal metamorphosis, while JH prevents premature metamorphosis and determines whether an ecdysteroid-induced molt is larva or pupa(Jindra et al., 2013; Jindra et al., 2015). In the adult stage, female reproduction, previtellogenic development, vitellogenesis, and oogenesis, in particular, are regulated by both 20E and JH (Sorge et al., 2000). In addition to hormones, the reproductive function of insects is also regulated by functional genes (Qian et al., 2020). Vitellogenin (Vg) is a high-volume protein synthesized in the fat body of female insects, after which oocyte maturation occurs (Yang et al., 2014). Vg is transported to the ovary by binding to the Vg receptor (VgR), a member of the low-density lipoprotein receptor (LDLR) family that is found on the oocyte membrane (Mitchell et al., 2019). The bound Vg is then transported into developing oocytes *via* mechanisms involving clathrin and Ras-like GTPase Rab protein (Tufail and Takeda, 2009; Tufail et al., 2014; Elmogy et al., 2018). The insect OVO protein encoded by the *ovo* gene belongs to the zinc finger protein family, which plays an important role in the growth and development of organisms (Piloto and Schilling, 2010; Lapan and Reddien, 2012). It is required in the female germ line for proper oogenesis and is necessary for the development of germ cells (Xing et al., 1988; Zhu et al., 2019). In germ cells, OVO binds directly to the promoter of the ovarian tumor (out) gene to regulate its expression and the development of ovary and germ cells (Hinson and Nagoshi, 1999; Xue et al., 2014). Both the *ovo* and *out* genes have been implicated in the regulation of the formation of female germ cells during early and late stages (Hinson et al., 1999). Sex-lethal (Sxl), an RNA-binding protein, is required for the induction of the female sexual identity in both somatic and germline cells (Ota et al., 2017).

In this study, we evaluated the impacts of two host plants (corn and goosegrass) on the development and reproduction of *S. frugiperda*. We hypothesized that the different ovary sizes in the FAW, which feed on different host plants, are influenced by the suppression of ovary development-related genes in the FAW from feeding on the hosts. Thus, to understand the reproductive difference and the mechanism by which the FAW responds to host plants, we used Illumina deep sequencing approaches to compare changes in the ovary transcriptomes of the FAW fed on corn and goosegrass. Of the total of 11 214 genes identified, 881 (7.86%) were found to be differentially expressed in the ovary of the FAW fed on either corn or goosegrass. The differentially expressed genes (DEGs) were mainly categorized into those involved in the renin-angiotensin system, viral myocarditis, and hypertrophic cardiomyopathy. In total, nine JH-biosynthetic genes, four 20E biosynthetic genes, and four ovary-relevant functional genes were identified by RNA-seq, and half of these showed changes. These data provided a transcriptomic map or landscape of the FAW ovary that indicates an important shift in the mRNA contents of the ovary in response to host plants, providing clues that can link developmental/reproductive pathways to FAW growth and reproduction.

## Materials and methods

### Insect culture

The fall armyworm (FAW), *S. frugiperda* (J. E. Smith), used in these experiments was reared for eight generations on corn or goosegrass plants, in the Insect Molecular Ecology Laboratory of Anhui Agricultural University, Hefei, Anhui Province. Plant seeds were purchased from an agricultural company (Shouhe Co., Ltd., China). Plants were grown through the three to four true-leaf stage before being employed in the studies (Lv et al., 2021). They were housed in cages at a temperature of 25 ± 3°C, with a relative humidity of 70 ± 10 percent and a photoperiod of 16 h light and 8 h dark.

### Growth and development of *S. frugiperda* on different host plants

Newly hatched larvae were transferred to rearing cages until the third instar stage, after which they were placed in 12-well acrylic plates (Biosharp^®^, Labgic Technology Co., Ltd., China). Larval and adult development were observed and recorded daily (60 larvae and 30 adults for each group) for the FAW raised on the two plants. The pupal weights for the two FAW groups were also recorded (30 pupa for each diet); the second day pupae were weighed on an ME203E analytical balance (Tuoliduo Co., Ltd., China) to a precision of 0.001 g. The females mated with males at 3-day post-eclosion for 24 h. The ovaries of 1- and 3-day post-eclosion were collected from virgin females, and the ovaries of 5-, 7-, and 9-day post-eclosion were from mated females. The lifespan of females and males was observed in a group. The number of eggs laid was examined at 9-d post-eclosion.

### Sample anatomy and collection

Samples of FAW adults aged 1, 3, 5, 7, and 9 days after eclosion were collected for the experiment. Using anatomy scissors, the legs, elytra, and metathoracic wings were first removed. Then, a live female or male was fixed on a wax-coated dish containing phosphate-buffered saline, using a microscope (SMZ1500, Nikon Inc, Japan). The FAW body was cut gently along the midline after fixing on the wax-coated dish. The internal reproductive organ of an adult FAW was removed by grasping the abdominal end with forceps and pulling it horizontally. They were either placed on glass slides or into new centrifuge tubes containing phosphate-buffered saline (Guo et al., 2019). The reproductive organs were examined and photographed using a stereomicroscope (Nikon Eclipse 80i, Nikon Inc, Japan). The other collected samples were frozen in liquid nitrogen.

### Transcriptome sequencing and analysis

The collected ovaries were homogenized in TRIzol reagent (Invitrogen, United States), and total RNA was extracted, following the manufacturer’s protocol. RNA purity was determined using a NanoDrop 2000 spectrophotometer (NanoDrop Technologies; United States), and the RNA integrity was verified using 1.5% agarose gels. Magnetic beads conjugated with oligo (dT) were used to isolate mRNA, which was fragmented into short lengths of approximately 200 bp using an RNA fragmentation kit (Illumina, United States) (Lin et al., 2018). Transcript library construction and sequencing were performed by Shanghai Majorbio Bio-pharm Biotechnology Co., Ltd. (Shanghai, China). After getting the raw sequence, adapter sequences and low-quality reads were removed to get clean reads. Once the raw reads were filtered, the *de novo* assembly of the FAW transcriptome was mapped to *S. frugiperda*’s genome database (Xiao et al., 2020). Differentially expressed gene (DEG) analysis of ovaries was carried out using DESeq2 (Love et al., 2014). After that, Gene Ontology (GO) (available online: http://geneontology. org/) and Kyoto Encyclopedia of Genes and Genomes (KEGG) analyses were performed, and significant enrichment was determined at *p* < 0.05.

### Gene expression analysis

The list of primers used in this study is given in Supplementary Table S1. Total RNA was extracted, according to the manufacturer’s protocol. Samples of cDNA were reverse-transcribed using 2 μg total DNase-treated RNA in a 20 μL reaction using the PrimeScript TM RT reagent kit (TaKaRa, China). RT-qPCRs were conducted using the CFX96TM real-time system (Bio-Rad, Hercules, CA, United States) with SYBR Green (TaKaRa, China) using the following cycling parameters: 95° C for 3 min, and 40 cycles of 95° C for 5 s, 60° C for 15 s, followed by a melting curve generation from 65° C to 95° C. All protocols for RT-qPCR experiments were in accordance with the minimum information required for publication of quantitative real-time PCR experiment guidelines (Bustin et al., 2009).

### Statistical analysis

The test data are systematically counted by Excel, and the statistical results are presented as mean ± SEM, and the *p*-values were calculated using the Mann–Whitney *U* test provided by SPSS 23.0 software. Other methods for calculating significance are specified in the figure legends.

## Results

### Host plants impact development and reproduction in the fall armyworm

**FIGURE 1:**
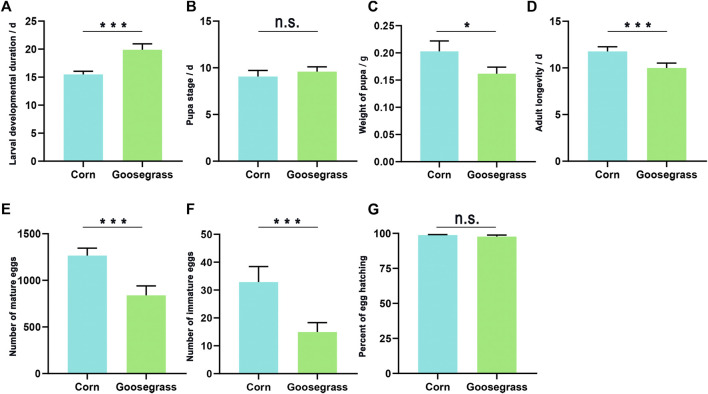
Biological parameters of *S. frugiperda* fed on corn and goosegrass. **(A)** Larval developmental duration. **(B)** Pupal stage. **(C)** Weight of the pupa. **(D)** Adult longevity. **(E)** Number of mature eggs. **(F)** Number of immature eggs. **(G)** Percent of egg hatched. Asterisks indicate significant differences between hosts (*, *p* < 0.05; **, *p* < 0.01, ***, and *p* < 0.001t).

The developmental periods for the immature stage, pupal weight, adult longevity, female fecundity, and egg hatching rate of the FAW, which fed on corn and goosegrass, are shown in Figure 1. The duration of the larval stage of the FAW reared on goosegrass (19.88 days) was significantly higher than that reared on corn (15.49 d) (*p* < 0.001). The pupal weight of the FAW fed on goosegrass (0.16 g) was significantly lower than that fed on corn (0.20 g) (*p* = 0.004). However, the duration of the pupal stage of the FAW which fed on corn (9.07 d) was not significantly different from that which fed on goosegrass (9.58 d). Adult longevity (both female and male) was significantly longer on corn (11.77 d) than that on goosegrass (9.99 d) (*p* = 0.001). The number of eggs laid by females, differed markedly among the two host plants (*p* < 0.001), with the recorded number being 1,265.92 on corn and only 839.82 on goosegrass. The number of residual eggs was still significantly different with 32.85 recorded on corn and 14.95 on goosegrass. The rate of egg hatching on corn and goosegrass was similar; nearly a 100% hatching rate was recorded on the two plants.

**FIGURE 2:**
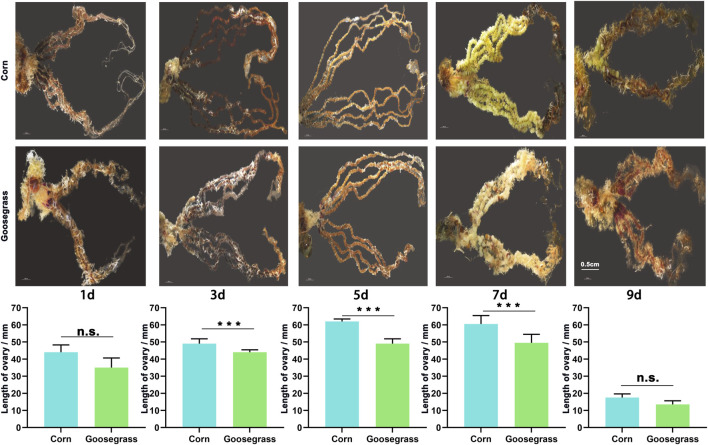
Effects of corn and goosegrass on the sizes of ovaries in *S. frugiperda*.

To further evaluate the impact of the different host plants on gonadal development, we dissected the testes and ovaries from FAW males and females, respectively. The ovarian development and size that fed on goosegrass were diminished at various adult stages (Figure 2). At 1-day post-eclosion, females were not mated, ovarian egg cells were not matured, and the follicle was visible. The mean length of the ovaries of the FAW which fed on corn was 44.63 mm and that on goosegrass was 39.84 mm, but the difference was not significant (*p* = 0.086). At 3 days post-eclosion, a small number of egg cells near the fallopian tube showed maturity, and the length of the ovaries of the FAW which fed on corn (49.44 mm) was significantly higher than that on goosegrass (43.23 mm) (*p* < 0.01). When the adult emerged 5 days later, most of the egg cells in the ovary showed fair maturation and appeared as yellow–green plump globules. The average length of ovaries of the FAW which fed on corn was significantly higher than that on goosegrass (*p* < 0.01). At 7 days post-eclosion, an important oviposition period, the mean length of the ovaries of the FAW which fed on corn (59.17 mm) was significantly higher than that on goosegrass (46.79 mm) (*p* < 0.01). At 9 days post-eclosion, most of the eggs were laid and ovaries were markedly atrophied, with their lengths greatly shortened. However, the difference between corn (16.12 mm) and goosegrass (13.22 mm) was not significant. The testis size also showed significant difference between corn and goosegrass at the early adult stages (1, 3, and 5 d post-eclosion) (Supplementary Figure S1). These results showed that goosegrass delayed gonadal development of *S. frugiperda*, compared to corn.

### Transcriptome sequencing and identification of differentially expressed genes

**FIGURE 3:**
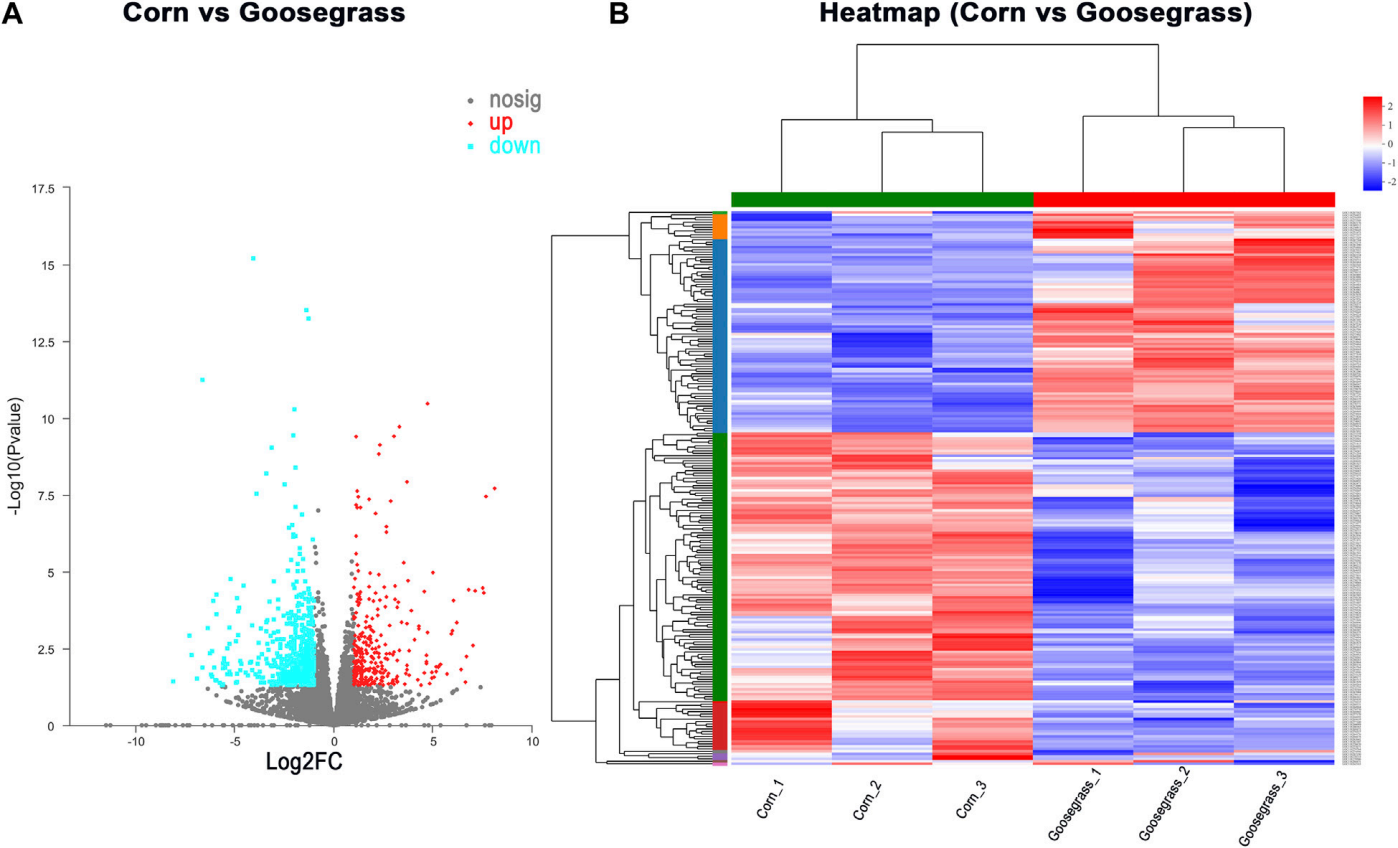
Overview of transcriptome data. **(A)** Volcano plot and **(B)** heatmap for all obtained genes.

**FIGURE 4:**
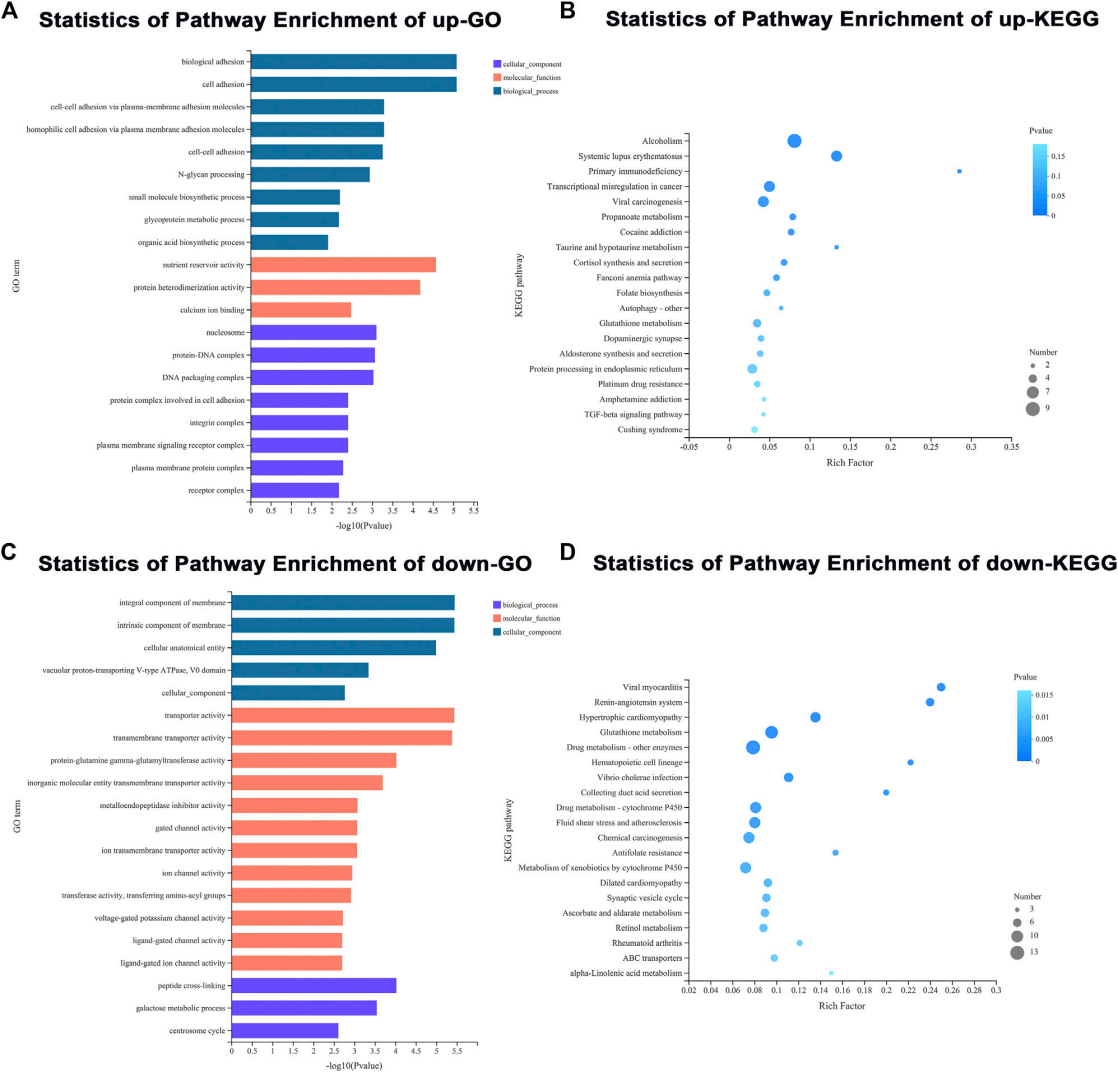
GO and KEGG enrichment analyses. **(A,B)** GO and KEGG pathway enrichment analyses of downregulated DEGs. **(C,D)** GO and KEGG pathway enrichment analyses of upregulated DEGs.

To gain insights into the underlying role of diet in ovarian development, an adult ovary was collected from the control and treatment groups at 3 d of eclosion for RNA-seq. A total of two RNA libraries (three replicates each group) were prepared and sequenced to a depth ranging from 45 615 164 to 57 987 112 total reads (Supplementary Table S2). In our RNA-seq, a total of 11 214 genes were identified in the ovary of an adult female FAW. Differentially expressed genes (DEGs) between goosegrass-reared populations and corn-reared populations were identified. Pairwise comparisons between the two different host plants were made with *p* < 0.05 and log2 fold-change ≥ 2 or ≤ −2 as cutoffs. A total of 881 (7.86%) DEGs were identified in the FAW ovary, including 300 significantly upregulated and 581 downregulated DEGs in the goosegrass groups compared to the corn groups (Figure 3A; Supplementary Table S3). Furthermore, a general overview of the expression pattern for the six groups was visualized using a heatmap (Figure 3B), providing an overall understanding of the changes in gene expressions. The differentially expressed genes were functionally annotated into biological process (BP), cellular component (CC), and molecular function (MF) by Gene Ontology (GO) analysis (Figure 4). Results showed that 72, 33, and 26 significantly-enriched GO terms under BP, CC, and MF categories, respectively, showed upregulation (Figure 4A; Supplementary Table S4). The number of GO terms which showed downregulation was 67, 21, and 62 under BP, CC, and MF categories, respectively (Figure 4B; Supplementary Table S5). The DEGs were mapped to reference pathways in the KEGG database to identify significantly enriched metabolic or signal transduction pathways. For upregulated DEGs, 10 pathways were significantly enriched, including “alcoholism,” “systemic lupus erythematosus,” and “primary immunodeficiency” (Figure 4C; Supplementary Table S6). For downregulated DEGs, 36 significantly enriched pathways were obtained; the top three identified pathways were “viral myocarditis,” “renin-angiotensin system,” and “hypertrophic cardiomyopathy” (Figure 4D; Supplementary Table S7).

### Important genes related to ovarian development

**FIGURE 5:**
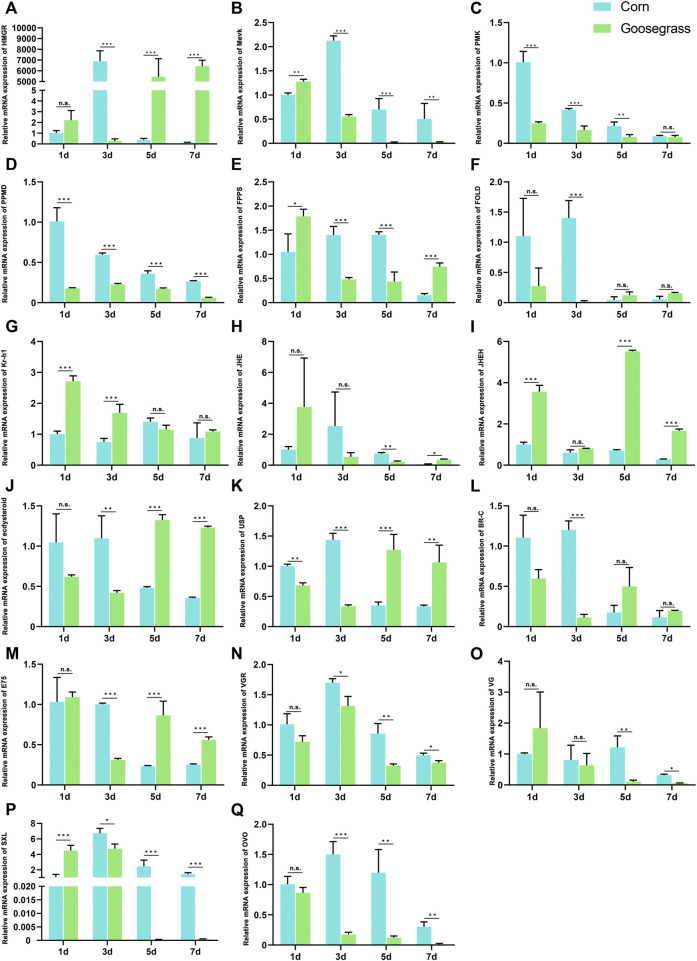
Relative expression levels of **(A–I)** JH-biosynthetic genes, **(J–M)** 20E-biosynthetic genes, and **(N–Q)** ovary development-related genes at 1, 3, 5, and 7 d post-eclosion determined by qRT-PCR. Asterisks indicate significant differences between hosts (*, *p* < 0.05; **, *p* < 0.01, ***; and *p* < 0.001).

Analysis of KEGG pathway analysis identified enrichment of the steroid hormone biosynthesis pathway in downregulated DEGs. To further determine whether different host plants influence the changes in the expressions of genes involved in FAW reproduction, 20E-biosynthetic, and JH-biosynthetic genes were further investigated. A summary of the distribution of hormonal biosynthetic genes and ovary-related genes in the FAW is given in Supplementary Table S8, including nine JH-biosynthetic genes, four 20E-biosynthetic genes, and four ovary-related genes. We measured their time course expressions using qRT-PCR (Figure 5). Expressions of the selected genes were prominent at the 7-d post-eclosion stage, due to the size of the ovary. They showed significant differences during the 3–7-d post-eclosion period. The genes, *Mevk, PMK, Vg, VgR, ovo,* and *Sxl* showed lower expressions in response to feeding on goosegrass, whereas *Kr-h1* and *JHEH* showed higher expressions. The other hormone-related genes showed no significant changes in their expressions.

## Discussion

As a highly polyphagous invasive pest, which has a strong capability of long-distance migration, *S. frugiperda* can damage different plant species, and it does not enter diapause (Du Plessis et al., 2020). Previous research studies mainly focused on the effect of chemical pesticides on the morphological characteristics of reproductive organs in *S. frugiperda* (Alves et al., 2014; Silva et al., 2016), but few studies have studied the effect of hosts on the gonad. Our study provided an overview of the developmental processes in FAWs fed on two different host plants, particularly on reproductive organ development. Goosegrass, an annual gramineous weed, is one of the major weeds in corn fields (Liu et al., 2022), which can become an alternative plant host for the FAW when corn is not available. The development of *S. frugiperda* populations, including developmental time, embryonic development, and the fecundity level, differed on the two different host plants. The FAW fed on goosegrass had a longer developmental duration and a reduced fecundity, which indicated that the goosegrass seriously repressed FAW’s growth and reproduction. Different host plants have been reported to have effects on the growth and development of herbivorous insects but, more importantly, also on their reproductive fitness (Li et al., 2016). To further evaluate the effects of feeding on different host plants on gonadal development, the ovarian and testicle sizes were measured at various adult stages. The length of the ovariole first showed an increasing trend, then later to a decreasing trend. The growth in the size of ovaries in the FAW fed on corn and goosegrass followed a consistent trend, which indicated that the two host plants provided nutrition for the normal growth of ovaries. However, the length of the ovaries of female adults fed on corn was generally longer than those fed on goosegrass before the end of the oviposition period. Our results showed that the length of ovary in the goosegrass group was decreased at 3, 5, and 7 d, by only 87.44%, 81.35%, and 79.08% of that in the corn group, respectively. These results suggested that corn provided a more suitable energy and nutrition for ovarian development in the FAW compared to goosegrass, which may have resulted in the difference in the ovarian size. We also found that the size of the testicle of the male FAW showed a decreasing trend, with an increasing age. However, the size of the testicle of the FAW fed on corn was significantly larger than that on goosegrass at the early adult stage, suggesting that diet was the main factor regulating the morphology of the male moth testis.

The detailed molecular mechanism underlying host plant regulation of the reproductive system in *S. frugiperda* is unclear. To globally analyze the effect of host plants on ovarian development, comparative transcriptomic analysis was performed using the ovaries of the FAW fed on corn and goosegrass with RNA-seq. We found that the FAW fed on goosegrass had 381 upregulated genes and 500 downregulated genes. GO terms of the DEGs relative to all genes were enriched in an integral component of membrane, intrinsic component of membrane, transporter activity, transmembrane transporter activity, and cellular anatomical entity, which provided important clues for understanding the effects of changes in gene expressions on FAW ovarian development. Insect reproduction is governed by two critical endocrines, JH and 20E (Gruntenko and Rauschenbach, 2008), which indicated that their biosynthetic genes play key roles in insect reproduction. Thus, we identified ten JH-biosynthetic genes and four 20E-biosynthetic genes. qRT-PCR analysis showed that the expressions of *Mevk* and *PMK* were lower and those of *Kr-h1* and *JHEH* were higher in the FAW fed on goosegrass. However, the 20E-biosynthetic genes showed no significant changes. *MevK*, *PMK,* and *Kr-h1* are associated with JH biosynthesis, while *JHEH* is related to JH degradation (Lu et al., 2015). These results indicate that host plants may influence a decrease in JH titers but may have no significant effect on the 20E titer. This indicated that the accurate regulation of JH titers in hemolymph is related to ovarian development.

Ovarian development and egg production in the FAW are also regulated by ovary development-related genes. We selected four-related genes, including *Vg*, *VgR*, *ovo*, and *Sxl*, from the ovarian transcriptomic data. We found that all these genes showed lower expression in the FAW fed on goosegrass than those fed on corn. Vg, the yolk protein precursor, is a high-volume protein synthesized in the fat body, transported by the hemolymph and deposited into maturing oocytes, a process governed by JH (Roy et al., 2018; Jing et al., 2021) which was also confirmed by our results. VgR functions as a transporter of Vg in female adults, which is essential for ovary development and oviposition (Matozzo and Marin, 2005). The *ovo* gene plays an important role in the growth and development of ovarian cells. Knockout of the *ovo* gene resulted in a significant reduction in the gonadal development (Bi et al., 2019). *Sxl,* the regulatory center of sex determination, also plays a critical role in female germ cells (Salz, 2013). These genes are, therefore, necessary to determine how the sizes of gonads are affected by host plants.

At present, there is no report on the effects of host plants on JH regulation and ovarian development in FAW. In this study, we evaluated the effects of two host plants on the growth, development, and reproduction in the FAW, particularly on the expression level of ovary development-related genes and effects on the mechanisms underlying 20E and JH coordinated regulation. These findings improved our understanding on how the host plant preference of *S. frugiperda* influences its reproductive activity. Additionally, they suggest how a new pest control strategy could be developed by genetically manipulating ovaries.

## Data Availability

The datasets generated for this study are available in the NCBI Sequence Read Archive under BioProject PRJNA886096 with the accession number SUB12117580.
