# Supplementary figures and images for: Comparative transcriptome analysis reveals key candidate genes mediating ovarian development in Spodoptera frugiperda fed on two host plants
Source: Front Physiol. 2022 Nov 15;13:1056540. doi: 10.3389/fphys.2022.1056540 (PMC9705327; doi:10.3389/fphys.2022.1056540)

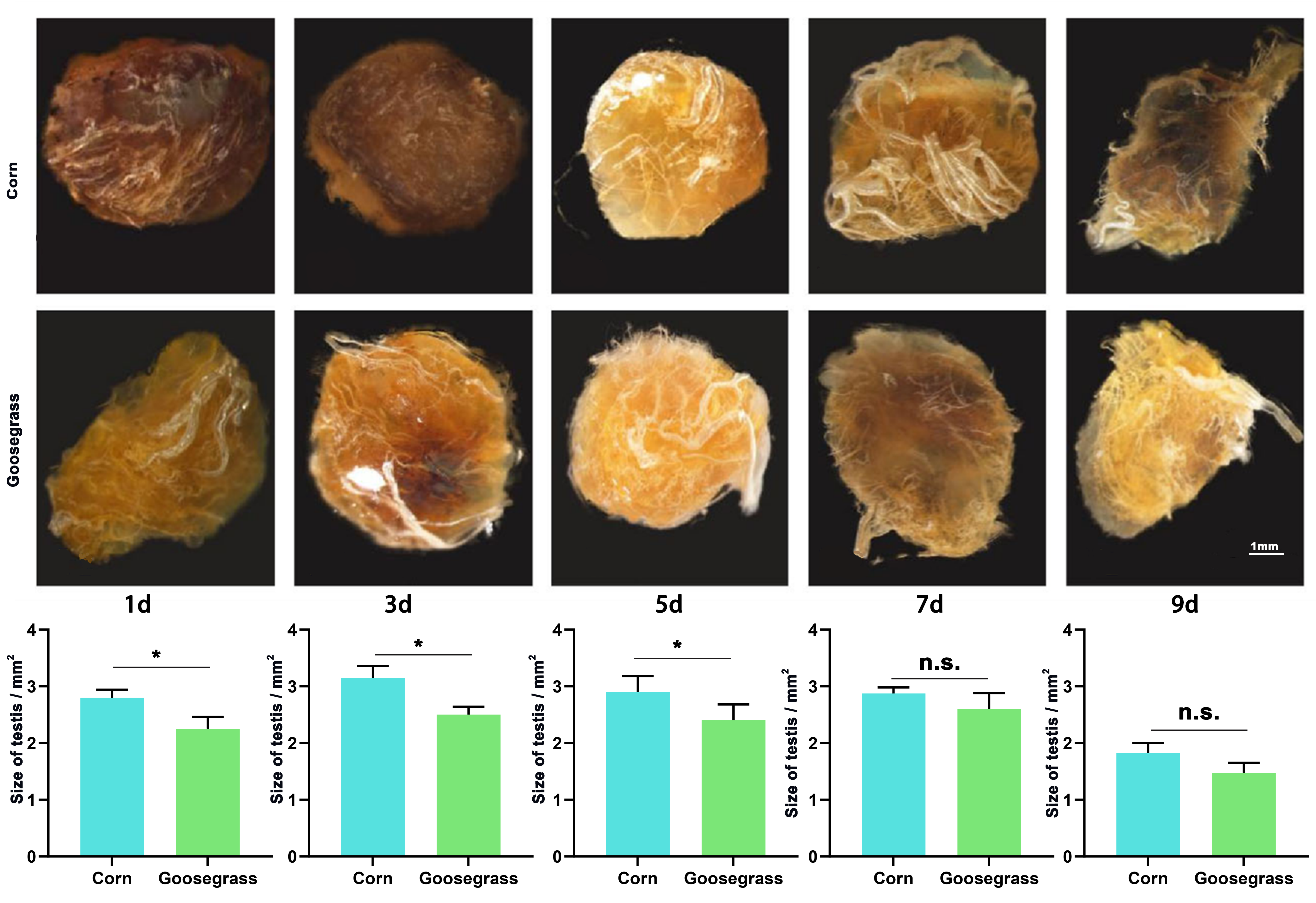

Supplement: Supplementary file 3 [file Image1.TIF]
